# Successful Treatment of Carcinomatous Central Airway Obstruction with Bronchoscopic Electrocautery Using Hot Biopsy Forceps during Mechanical Ventilation

**DOI:** 10.1155/2017/5378583

**Published:** 2017-03-08

**Authors:** Motoi Ugajin, Hisanori Kani

**Affiliations:** ^1^Department of Respiratory Medicine, Nagoya Tokushukai General Hospital, Aichi, Japan; ^2^Department of Thoracic Surgery, Nagoya Tokushukai General Hospital, Aichi, Japan

## Abstract

We report the case of a 72-year-old man with occlusion of the left main bronchus due to squamous cell carcinoma of the lung. He required tracheal intubation and mechanical ventilation because of the aggravation of atelectasis and obstructive pneumonia. Electrocautery using hot biopsy forceps was performed during mechanical ventilation with a 40% fraction of inspired oxygen. He was extubated following improvement in the atelectasis and obstructive pneumonia and discharged with shrinkage of the tumor after chemotherapy. We describe a safe electrocautery procedure using hot biopsy forceps during mechanical ventilation with reference to previous reports.

## 1. Introduction

Central airway obstruction can result from a variety of disease processes and is a significant cause of mortality [1]. Various interventional approaches have been developed for the treatment of central airway obstruction, such as the development of the bronchoscope. Regarding devices used with bronchoscopes in interventions for central airway obstruction, the Nd-YAG laser is most commonly used across the world. However, compared with Nd-YAG laser treatment for central airway obstruction, electrocautery has been reported to be equally useful [2]. We successfully treated a patient with carcinomatous central airway obstruction during mechanical ventilation with electrocautery using hot biopsy forceps.

## 2. Case Report

**Figure 1:**
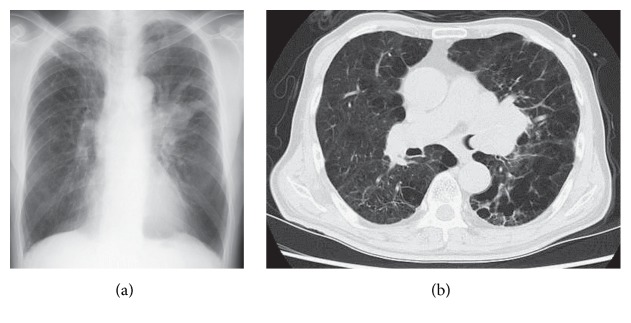
(a) Chest X-ray on admission revealed a mass in the left hilar region. (b) Chest computed tomography on admission showed a mass invading the left main bronchus.

**Figure 2:**
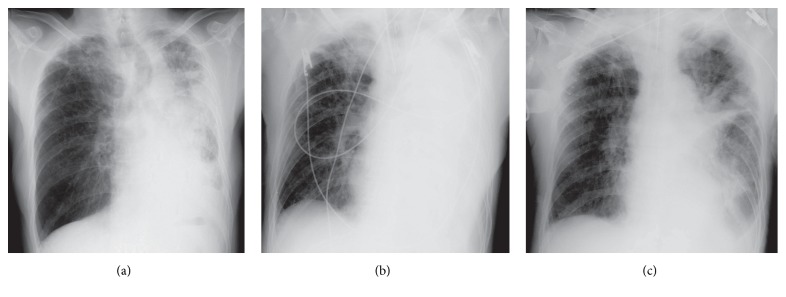
(a) Chest X-ray 3 days after admission revealed the progression of atelectasis in the left lung. (b) Chest X-ray on the day of intubation revealed complete atelectasis of the left lung. (c) Chest X-ray a day after the second electrocautery procedure showed marked improvement of the atelectasis of the left lung.

A 72-year-old man presented to the outpatient department with hemosputum and worsening dyspnea in August 2016. He regularly took tiotropium because of chronic obstructive pulmonary disease. His lung function test performed 8 months previously showed that his forced expiratory volume in 1 second (FEV_1.0_) was 1.49 L (53.0% of the predicted value) and FEV_1.0_% was 44.7%. He also had a regular prescription of aspirin and clopidogrel due to a history of ischemic heart disease. His chest X-ray on admission suggested a large mass in his left lung field (Figure 1(a)) and his chest computed tomography revealed a tumor invading the left secondary carina (Figure 1(b)). The diagnostic bronchoscopy was scheduled to be performed several days after the cessation of aspirin and clopidogrel. While waiting for the diagnostic bronchoscopy, he required tracheal intubation and mechanical ventilation due to atelectasis and obstructive pneumonia (Figures 2(a) and 2(b)).

**Figure 3:**
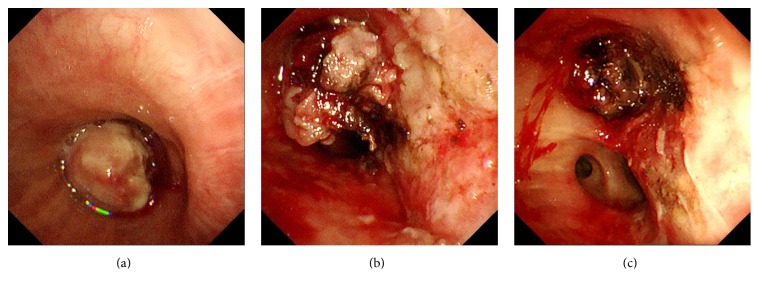
(a) A polypoid tumor completely occluded the lumen of the left main bronchus. (b) After the first electrocautery procedure, the left inferior lobar bronchus could be partially visualized. (c) After the second electrocautery procedure performed 4 days after the first one, the left inferior lobar bronchus could be fully visualized.

Diagnostic and interventional bronchoscopy using a flexible fiberscope (BF-1TQ290; Olympus Corporation, Tokyo, Japan) was performed during mechanical ventilation. Pressure-controlled setting was used for the ventilation and the fraction of inspired oxygen (FiO_2_) was set on 40% to maintain 90% oxygen saturation. Bronchoscopic examination showed that the left main bronchus was completely occluded by a polypoid tumor (Figure 3(a)). Electrocautery using hot biopsy forceps (Radial Jaw 4, Boston Scientific, MA, USA) was performed after transbronchial biopsy of the tumor. The high-frequency generator (ICC 350, ERBE, Tubingen, Germany) was set on coagulation mode with a maximum power of 40 W. Electrocautery was performed twice, once on the day of the biopsy and again four days later. The first and second electrocautery required 153 and 108 minutes, respectively. The patency of the left inferior lobar bronchus was restored without complication after electrocautery (Figures 3(b) and 3(c)). He was extubated following improvement of the atelectasis and obstructive pneumonia (Figure 2(c)). Pathological examination of the biopsy specimen revealed squamous cell carcinoma of the lung. Whole-body examination led to the squamous cell carcinoma being staged as cT4N2M0. Seven days after the second electrocautery, carboplatin (area under the curve of 4) and nab-paclitaxel (80 mg/m^2^) were administered as first-line chemotherapy due to his Eastern Cooperative Oncology Group performance status of 2. He was discharged with marked shrinkage of the tumor 26 days after the second electrocautery.

## 3. Discussion

The present report showed that the electrocautery using hot biopsy forceps is a potential useful technique to manage carcinomatous central airway obstruction. Previous studies have demonstrated that high-frequency wire snares are useful devices for bronchoscopic electrocautery [3, 4]. However, it is often difficult to apply a wire snare to wide-based tumor [5]. In the present case, the neck of the tumor could not be visualized and we could not pass a wire snare beyond the tumor due to complete occlusion of the lumen of the left main bronchus. Therefore, we decided to apply hot biopsy forceps instead of high-frequency wire snare for this case.

Tremblay et al. reported that hot biopsy forceps reduced the amount of bleeding related to biopsy without a negative impact on the pathological specimen [6]. Up to now, hot biopsy forceps have been recognized as a device for the biopsy of endobronchial lesions rather than an electrocautery device. As far as we know, there is only one case report of the use of hot biopsy forceps in endobronchial electrocautery [7]. In the present case, we could restore the patency of central airway after electrocautery using hot biopsy forceps without complication. Although long procedure time was required as compared to a previous report using argon plasma coagulation [8], hot biopsy forceps can be an option as an interventional device for central airway obstruction.

Regarding treatments for central airway obstruction, various interventional devices are available, including Nd-YAG laser, argon plasma coagulation (APC), cryotherapy, and electrocautery. Each device has its own advantages as well as disadvantages [9]. The Nd-YAG laser is currently the most established device [10]. However, it should not be used in high-oxygen environment which requires more than 40% FiO_2_ because of the high risk of ignition [9]. The APC is a noncontact mode of monopolar electrical coagulation which uses argon gas as the conductive media. The APC has been reported to be safe as electrocautery for interventional bronchoscopy [11]. However, similarly to the Nd-YAG laser, it is recommended to avoid using the APC in high-oxygen environment [5]. The cryotherapy can be applied in high-oxygen environment, but several days are required to show its effect. The present case required 40% FiO_2_ to maintain the essential oxygen saturation and early withdrawal from mechanical ventilation was indispensable to perform the following chemotherapy. Therefore, we decided to apply electrocautery for the management of central airway obstruction in the present case.

Access to electrocautery is typically much easier than that to the Nd-YAG laser. Although a high-frequency generator is a standard instrument in almost every endoscopy and surgical unit, Nd-YAG laser machines are not so common [2]. In fact, our hospital, which has 350 beds, an endoscopy unit, and eight operating rooms, does not have any Nd-YAG laser machines; in contrast, there are at least 10 available high-frequency generators. Sindhwani et al. reported that electrocautery for central airway obstruction was a safe and effective technique in a hospital where access to laser and cryotherapy was limited [12]. Due to its easy access and cost-effectiveness, electrocautery has been a more popular device for interventions in central airway obstruction [13].

Ignition is one of the major problems caused by electrocautery. There are a considerable number of reports that electrocautery can cause fires and significant negative outcomes for patients [14–16]. The possibility of ignition caused by electrocautery is related to the inspired oxygen concentration. Consequently, the use of the minimum inspired oxygen concentration with satisfactory oxyhemoglobin saturation is recommended [17]. However, a definitive inspired oxygen concentration for safe electrocautery has not yet been established. In the present case, 40% FiO_2_ was required to maintain the essential oxygen saturation. We performed endobronchial electrocautery without complication during mechanical ventilation with a 40% FiO_2_. The present case may provide a benchmark concerning inspired oxygen concentrations that are safe for endobronchial electrocautery.

Bronchial wall damage is known as a complication due to electrocautery. Long duration of electrocautery caused damage to the underlying cartilage, while short duration within 2 seconds of electrocautery brought only superficial damage [18]. Therefore, to avoid airway perforation, short bursts of energy within 2 seconds per pulse are recommended [19]. In terms of bronchial wall damage, hot biopsy forceps seems to be safer than high-frequency wire snare because forceps can handle a target without contact to bronchial wall. In the present case, we restrained hot biopsy forceps from touching bronchial wall as much as possible. When touching bronchial wall was inevitable, burst of energy was limited as short as possible.

In conclusion, electrocautery using hot biopsy forceps can be a therapeutic option for patients requiring mechanical ventilation due to carcinomatous central airway obstruction.
